# Glycerol Assisted Pretreatment of Lignocellulose Wheat Straw Materials as a Promising Approach for Fabrication of Sustainable Fibrous Filler for Biocomposites

**DOI:** 10.3390/polym13030388

**Published:** 2021-01-26

**Authors:** Hamayoun Mahmood, Saqib Mehmood, Ahmad Shakeel, Tanveer Iqbal, Mohsin Ali Kazmi, Abdul Rehman Khurram, Muhammad Moniruzzaman

**Affiliations:** 1 Department of Chemical, Polymer & Composite Materials Engineering, University of Engineering & Technology, New Campus, Lahore 54890, Pakistan; engr.hamayoun@uet.edu.pk (H.M.); saqibmhmod@gmail.com (S.M.); tanveer@uet.edu.pk (T.I.); drkazmi@uet.edu.pk (M.A.K.); abdulrehmankhurram415@gmail.com (A.R.K.); 2 Department of Hydraulic Engineering, Faculty of Civil Engineering and Geosciences, Delft University of Technology, Stevinweg 1, 2628 CN Delft, The Netherlands; 3 Department of Chemical Engineering, Universiti Teknologi Petronas, Bandar Seri Iskandar, Perak 32610, Malaysia; m.moniruzzaman@utp.edu.my

**Keywords:** lignocellulosic biomass, glycerol pretreatment, biocomposite board, energy consumption

## Abstract

Glycerol pretreatment is a promising method for the environmentally-friendly transformation of lignocellulosic materials into sustainable cellulose-rich raw materials (i.e., biopolymer) to fabricate biocomposites. Here, a comparison of aqueous acidified glycerol (AAG) pretreatment of wheat straw (WS) with alkaline, hot water, and dilute acid pretreatments on the thermal and mechanical characteristics of their fabricated composite board is presented. A comparison of total energy expenditure during WS pretreatment with AAG and other solutions was estimated and a comparative influence of AAG processing on lignocellulosic constituents and thermal stability of WS fiber was studied. Results imply that AAG pretreatment was superior in generating cellulose-rich fiber (CRF) as compared to other pretreatments and enhanced the cellulose contents by 90% compared to raw WS fiber. Flexural strength of acidic (40.50 MPa) and hot water treated WS composite (38.71 MPa) was higher compared to the value of 33.57 MPa for untreated composite, but AAG-treated composites exhibited lower values of flexural strength (22.22 MPa) compared to untreated composite samples. Conversely, AAG pretreatment consumed about 56% lesser energy for each kg of WS processed as compared to other pretreatments. These findings recognize that glycerol pretreatment could be a clean and new pretreatment strategy to convert agricultural waste into high-quality CRF as a sustainable raw material source for engineered biocomposite panels.

## 1. Introduction

Global development and quest for biodegradable materials have increased over the past few decades owing to public concern about the environment, depletion of fossil reserves, political commitment and climate change. To realize the UN climate panel target of 50–80% reduction in greenhouse gas emissions, the shift from bondage of petro-fuels to sustainable resources is indispensable. Lignocellulosic materials offer an alternative, abundant, renewable, environmentally-benign, and low-cost raw fiber source for manufacturing of a plethora of biobased products. The use of waste lignocellulosic fibers for biocomposites promotes the biorefinery concept with the vision of a sustainable economy (bioeconomy). About 140 billion tons of lignocellulosic biomass is generated annually worldwide and a significant part of it has been considered as waste with an absence of any conflict with food availability [1]. Biocomposites manufactured from agricultural waste exhibit less environmental impact in comparison with the wood particle-based biocomposites primarily due to carriage distance [2].

However, the incorporation of lignocellulosic fibers into the polymer matrix phase for composite manufacturing is challenging due to non-cellulosic constituents present over the fiber’s surface which impart weak fiber-binder interfacial conglutination [3]. Conversely, high susceptibility to thermal degradation is another ambitious problem during the development of lignocellulosic fiber based composites during manufacturing and service [4]. The presence of non-cellulosic impurities offers significant technological hindrance to the processing and widespread practical window-use of biocomposites. Numerous pretreatment strategies, including chemical [5], physical [6], hydrothermal [7], and biological [8], have been practiced to address the aforementioned problems by extraction of cellulose-rich fiber (CRF) from lignocellulosic materials that could have better dispersibility and adhesion with polymer matrix phase during thermal molding. Chemical pretreatments employ strong acids, bases, or ionic liquids to modify the structure and chemical attributes of lignocellulosic materials. Physical methods refer to techniques that do not use chemicals or enzymes during pretreatment processes such as comminution, extrusion and steam explosion. In hydrothermal methods, fiber is cooked in liquid water at elevated temperatures under high pressure [9,10].

Most of these fiber-cooking processes operate under extreme conditions of temperature and pressure or employ highly concentrated chemicals thus evoke critical environmental hazards and human health issues. The sustainable development goals (SDGs) of the UN in 2015 focus on numerous issues, many of which highlight the need for green and sustainable solvents for processing of renewable resources. Among various methods, organosolv pretreatment using glycerol can be conducted under normal pressure due to the inherent advantages of negligible volatility and economic benefits. Organosolv lignocellulose processing is realized as an emerging technology in a biorefinery context due to its advantages such as the ability to disintegrate lignocellulose into high-purity biopolymer components (cellulose, hemicellulose, and lignin) with prominent efficiency, low capital investment, and the possibility of executing it in small-scale units, as well as facile solvent recovery and reuse [11]. The high boiling point of glycerol offers low demands on temperature and pressure but could boost energy expenditure for solvent recovery [12]. The excellent dissolution ability of glycerol for organic materials recognizes them as a highly promising alternative to many ionic liquids which are proved to be toxic, exhibit poor bio-compatibility, and high cost [13]. Partial removal of lignin and hemicellulose as a result of glycerol pretreatment primarily contribute to enhanced accessible surface area of cellulose fibrils. This could provoke better fiber–matrix interaction during molding process in biocomposite manufacturing [14]. As a byproduct of the growing biodiesel industry, glycerol production has increased significantly in many parts of the world and its price has declined to as low as US $0.11/kg [15]. Sun et al. reported that atmospheric glycerol pretreatment significantly modified the physiochemical characteristics of sugarcane bagasse towards improved susceptibility to cellulase enzymes achieving 90% hydrolyzability in 72 h [16]. Trinh et al. noted that glycerol pretreatment of rice straw improved the digestibility of fermentable sugars by 4–5 times compared to that of untreated residue under 190 °C and 10 h pretreatment conditions [17]. Other studies can also be found in the literature showing excellent performance of glycerol organosolv pretreatment for biofuel production from lignocellulose [18,19]. However, to the best of our knowledge, use of glycerol pretreatment has yet to be considered for manufacturing of polymeric composites for lignocellulosic-based biorefineries. In this work, the potency of acidified aqueous glycerol (AAG) pretreatment is compared with prominent pretreatment techniques such as alkaline, dilute acid, and hot water for wheat straw pretreatment, prior to prepare composite panels with polyester resin as a matrix phase using thermal press molding. The impact of glycerol and other pretreatments on physicochemical properties of the fiber was also studied. A comparative impact of AAG pretreatment on the thermal and mechanical characteristics of the composite panels was conferred.

## 2. Materials and Methods

Wheat straw strands and polyester resin (SIR-8340) were kindly acquired from Fiber Craft Industries, Lahore, Pakistan. Resin SIR-8340 is a general purpose unsaturated polyester resin with styrene monomer, produced by Saudi Industrial Resins Ltd. Jeddah, Saudi Arabia. Glycerol and all other chemicals were of laboratory scale with purity >95% and used as received.

### 2.1. Pretreatment of Wheat Straw

Lignocellulosic wheat straw (WS) was ground, sieved (particle size ≤ 0.5 mm), and washed with distilled water to remove any dust particles. For pretreatment, dried wheat straw was added into aqueous acidified glycerol (1.2% HCl, 78.8% glycerol and remaining water) with 10% solid loading in a 500 mL conical flask [20]. Pretreatment was conducted using a paraffin oil bath under 130 °C and 30 min with a condenser and reflux arrangement to avoid loss of solvent. Besides, wheat WS fiber pretreatment with alkaline (NaOH 2% *w*/*w*), dilute acid (H_2_SO_4_ 1.82% *w*/*w*), and hot water was performed under similar conditions as those of AAG pretreatment under 10% fiber loading [21]. In each case, the pretreated cellulose-rich meal was washed with plenty of distilled water and dried at 70 °C overnight in an air-circulating oven.

#### 2.1.1. Estimation of Energy Expenditure for WS Pretreatment

Assuming the biomass pretreatment reactor as a closed system for which general thermodynamic energy balance equation can be written as [22]:

(1) $$∆U=Q+W+∆E_{k}+∆E_{p}$$

where Q is the thermal energy provided to the system; ∆U, $∆E_{p}$, and $∆E_{k}$ represent the internal, potential and kinetic energy changes, respectively. W represents the shaft work in the form of mechanical agitation. The terms for changes in potential and kinetic energies can be excluded owing to the absence of any accelerating or falling components,

(2) ∆U=Q+W .

Work done (W) by mechanical agitation can be calculated by estimating the power needed to move the impeller, which is obtained by multiplying the specific kinetic energy of the liquid ($E_{k}$) with the flow produced by the impeller (q) [23]:

(3) $$\;q=N\cdot D_{a}^{3}\cdot N_{Q}$$

(4) $$E_{k}=\rho \cdot v_{2}^{2}$$

where $D_{a}$ and N denote the diameter and speed of impeller, respectively, $N_{Q}$ is the Flow number, ρ is the density of pretreated mixture, and *v*_2_ is the flow velocity. Thus,

(5) $$P=N\cdot D_{a}^{3}\cdot N_{Q}\cdot \rho {\left(\alpha \cdot \pi \cdot n\cdot D_{a}\right)}^{2}/2$$

where α is the ratio *v*_2_/*u*_2_ in which *u*_2_ is the tip velocity. The following relation can be obtained from Equation (5) by applying the dimensional analysis:

(6) $$P=N^{3}\cdot D_{a}^{5}\cdot N_{P}$$

where $N_{P}$ designates the Power number, which is a function of physical characteristics of the pretreated mixture, flow pattern (laminar or turbulent), and the various shape factors.

Thermal energy supplied for the pretreatment of WS fiber can be obtained by using the following expression:

(7) $$Q=m\int_{T_{i}}^{T_{f}}C_{p}dT$$

where $T_{i}$ and $T_{f}$ are symbols for initial and final temperature, m is the mass and $C_{p}$ represents the heat capacity of the pretreatment mixture [24]. Thus, Equation (2) can be re-written to predict the total energy required to pretreat the wheat straw:

(8) $$Pretreatment\;energy=N^{3}\cdot D_{a}^{5}\cdot N_{P}+\;m\int_{T_{i}}^{T_{f}}C_{p}dT$$

#### 2.1.2. Lignocellulosic Composition Analysis

Contents of lignocellulosic constituents of untreated and pretreated WS samples were determined by the method reported elsewhere [25]. Briefly, cellulose contents were measured using a reagent containing acetic acid and concentrated nitric acid in a ratio of 10:1. Dried WS sample (0.1 g) was cooked in 3.0 mL of this reagent in a properly sealed test tube using a water bath for 30 min. After completion, mixture was diluted with distilled water and residue was separated using vacuum filtration, dried and weighed. Holocellulose contents of untreated and pretreated wheat straw samples were measured via treatment with an acidified sodium chlorite solution. The combined cellulose and hemicellulose contents are referred to as holocellulose. Thus, difference of holocellulose and cellulose will provide hemicellulose. Finally, lignin fraction was found conforming a two-step acid hydrolysis method using a 72% (*w*/*w*) solution of H_2_SO_4_.

#### 2.1.3. Thermogravimetric Analysis (TGA)

The thermal degradation profiles of untreated and treated WS samples were measured from room temperature to 600 °C using TGA (Model: SDT Q600) under heating rate of 10 °C/min with nitrogen flow rate of 20 mL/min.

### 2.2. Wheat Straw Cellulose-Rich Fiber-Filled Biocomposite Boards

**Figure 1:**
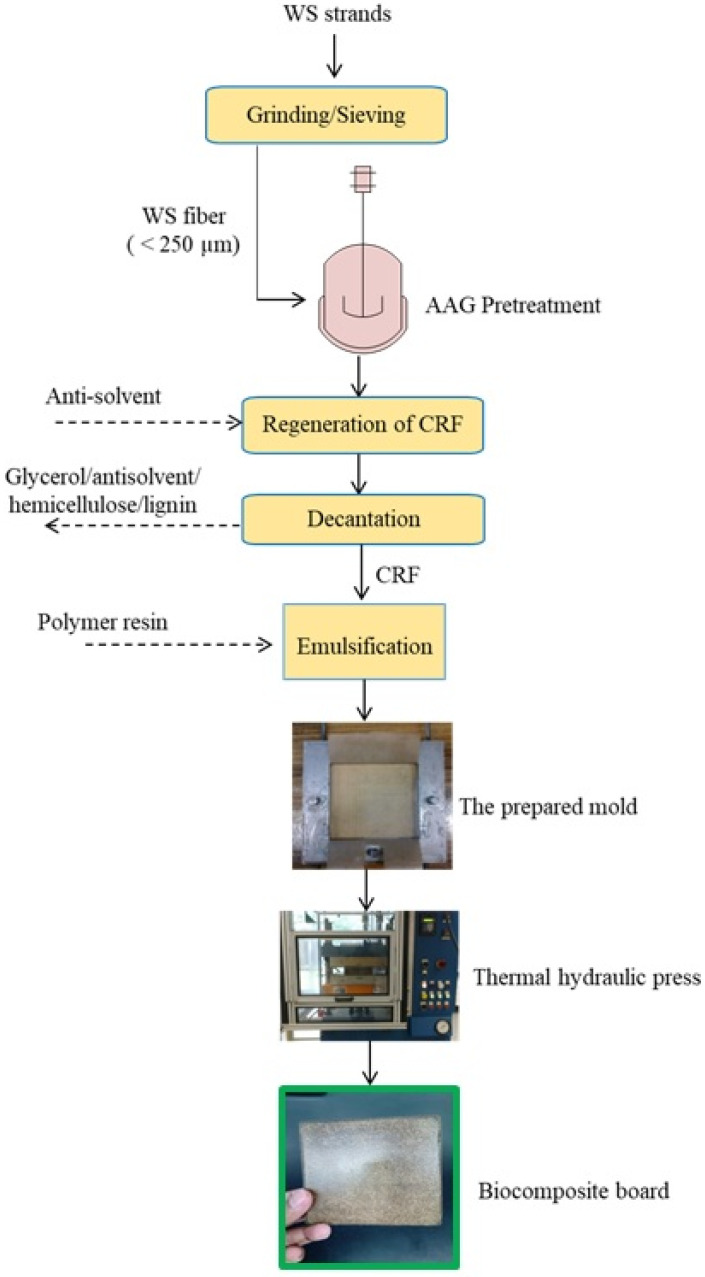
Schematic representation for the preparation of biocomposites from pretreated wheat straw (WS) fibers.

Polyester resin (SIR-8340) was used as a polymer matrix which was reinforced with WS particles under 25% (*w*/*w*) solid loading [26]. Typically, in a 60 g sample of polymer, accelerator (cobalt octoate, 2% *w*/*w*) was added and mixed thoroughly followed by addition of untreated or treated biomass fiber. Subsequently, the initiator (methyl ethyl ketone peroxide) was introduced dropwise in the matrix-fiber mixture under vigorous mixing and gel-like mixture was put into stainless steel mold and thermally pressed at 70 °C and 20 MPa for 10 min. Figure 1 portrays the various processing steps in the preparation of biocomposite samples from pretreated WS fibers.

#### 2.2.1. Mechanical Testing

After manufacturing, all biocomposite samples were stored in air-tight bags to avoid moisture absorption from environment. The three-point bending test (flexural mode) of the composite samples was performed on a universal testing machine (Model: Tira test 2810) using composite sample with 4 mm thickness and 40 mm gauge length. The cross-head speed for the test was 1 mm/min following ASTM-D790 standard [27].

#### 2.2.2. Thermogravimetric Analysis (TGA)

The thermal properties of untreated and composite panels made from various pretreated fibers were characterized by thermogravimetric analysis (Model: SDT Q600) under nitrogen atmosphere in the temperature span of 25–600 °C at the rate of 10 °C/min.

## 3. Results and Discussion

### 3.1. Lignocellulosic Characterization of WS Fibers

**Figure 2:**
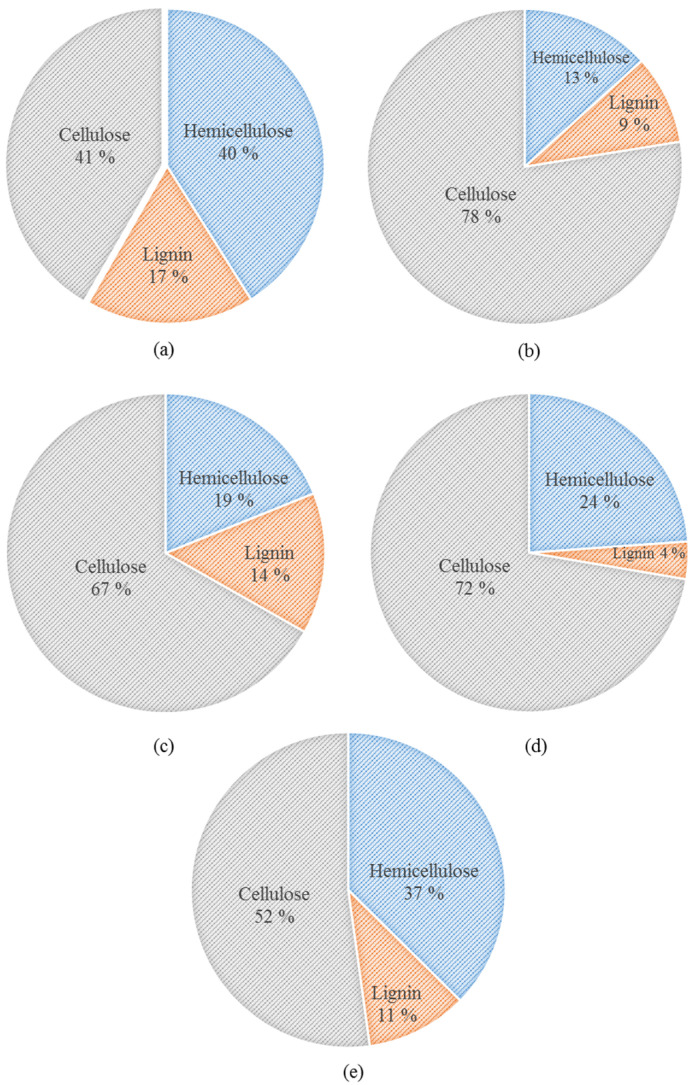
Comparison of lignocellulosic composition of untreated and after numerous pretreatments of wheat straw fiber: (**a**) Untreated, (**b**) glycerol pretreatment, (**c**) acid pretreatment, (**d**) alkali pretreatment, and (**e**) hot water pretreatment.

Figure 2 summarizes the results of lignocellulosic composition analysis of WS fibers for untreated and after numerous pretreatments as described in Section 2.1. Glycerol pretreatment showed superior capability to change the composition of WS fibers compared to other chemicals and enhanced the cellulose contents to 78% as compared to cellulose contents of 41% in raw WS fibers. Dilute acidic and alkali pretreatments also considerably increased the cellulose contents to 67% and 72%, respectively whereas hot water pretreatment was not much effective to produce cellulose-rich fibers from raw WS fibers. Likewise, acidified glycerol exhibited profound expediency for delignification of WS fibers than those of other solvents, except alkali, by eliminating around 47% of lignin present in the untreated WS fibers. Pretreatment using alkaline, acid, and hot water reagents provoked 77%, 18.2%, and 38.2% delignification from raw WS biomass, respectively.

**Figure 3:**
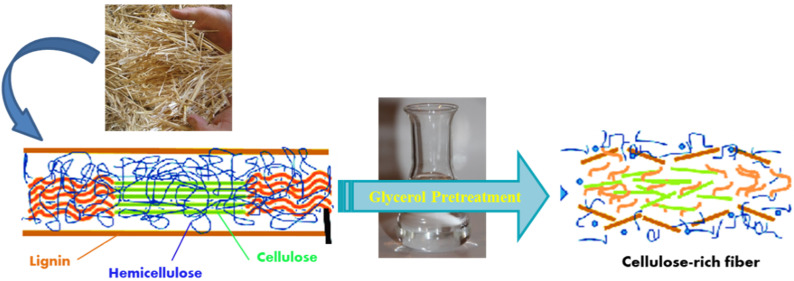
Schematic representation for acidified glycerol assisted generation of cellulose-rich fibrous material from waste wheat straw.

Glycerol pretreatment also significantly reduced the hemicellulose contents from 40% in raw fiber to 13%. Enhancement in the cellulosic contents by partial removal of hemicellulose and lignin after acidified pretreatment of different lignocellulosic biomass has been reported in the literature [16,18]. In the comparative study of Yu et al. [21], acid pretreatment removed 98% of hemicellulose and 5.4% of the lignin from corn stover, whereas alkali solution was found superior for lignin removal, which is in accordance with our results. The decrease in hemicellulose and lignin fractions may indicate that these components were dissolved in the AAG and other solvents. The dilute acid and alkali pretreatments extracted relatively high caliber CRF from raw WS fibers. Nevertheless, the principal recalcitrant constituent for the production of composites from agricultural fiber is lignin; in this regard, AAG pretreatment offered better competence in delignification, as illustrated in Figure 2. Figure 3 portrays a schematic for generation of cellulose-rich fibrous material from waste wheat straw by using acidified glycerol pretreatment.

### 3.2. Thermal Stability of WS Fibers after Glycerol-Based Pretreatment

**Figure 4:**
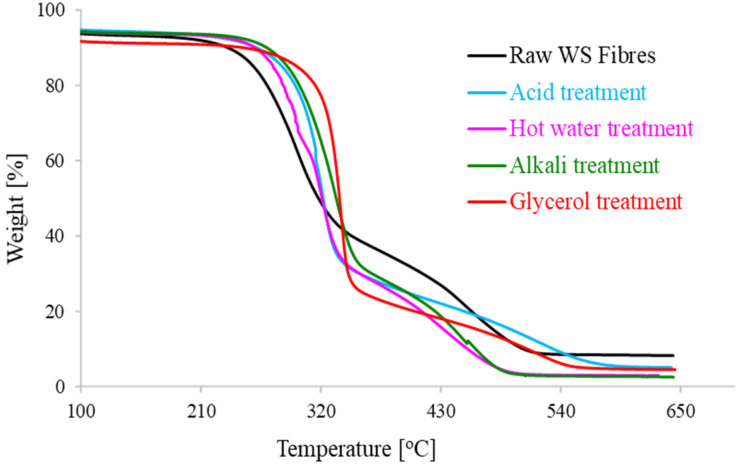
Thermal degradation profiles for untreated (raw) and treated WS fibers.

One of the major limitations of lignocellulosic fibers for compounding with various thermoplastic polymer matrices to prepare biocomposites is their lower thermal stability. Figure 4 shows the thermogravimetric and derivative thermogravimetric profiles for untreated WS fiber and after different pretreatments, while Table 1 summarizes various parameters inferred from these thermal profiles. The dilute acid, alkali and hot water solutions provoked the WS fiber with *T*_p_ (i.e., temperature of maximum thermal degradation) values of 315.8 °C, 333.1 °C and 313.2 °C compared to the value of 338.4 °C for glycerol treated WS fibers. The highest value of *T*_p_ shows the superiority of glycerol pretreatment in producing CRF as also indicated from lignocellulosic composition analysis (Figure 2). The fastest thermal disintegration occurs between temperature 275 to 400 °C which is mainly attributed to the degradation of cellulose. The long tail in TGA profiles after 500 °C corresponds to final degradation of stronger aromatics bonds of lignin, ultimately leading to the formation of a char [28]. Further, comparing at 600 °C, the values of residue left for glycerol, dilute acid, and alkali pretreatments were 4.9%, 5.9%, and 3.4%, respectively, which was lower in comparison with a value of 9.1% for raw WS fiber.

The transformation in thermal attributes of WS fibers after numerous pretreatments might be related to the alteration in lignocellulosic proportions of WS fiber [29]. All solvents partly removed hemicellulose and lignin from the raw WS samples. Actually, each biopolymer component of lignocellulose is acute to thermal demolition in a specific temperature span. Non-cellulosic constituents (hemicellulose and lignin) hold relatively weak resistance to thermal degradation compared to that of cellulose [30]. Lignocellulose thermal degradation profile is significantly affected by the change in lignocellulosic compositions and even can be an alternative rapid tool to estimate biomass composition. Particularly, hemicellulose has the lowest degradation temperature and degrades at a lower temperature compared to cellulose and lignin. Cellulose is the most thermally stable biopolymer with the highest decomposition temperature (around 320 °C) whereas lignin disintegrates over a broader temperature span [31]. The tightly arranged and ordered cellulose nanofibrils make the cellulose comparatively unreachable, providing it strength, stability and resistance to thermal decomposition [32]. An increase in cellulose contents (and consequently decrease in hemicellulose and lignin percentage) increases the peak temperature, leading to a higher reactivity of lignocellulosic fiber for interaction with polymer matrix. The comparative examination of thermal solidity of WS fibers after AAG pretreatment with different pretreatments imply that glycerol could be an effective and environmentally-benign pretreatment solvent to produce high-quality cellulosic fiber from agricultural waste.

### 3.3. Thermal Properties of WS-Derived Fibers Filled Biocomposite Boards

Sufficient resistance to thermal deterioration of biocomposites is indispensable to sustain their distinct properties at service temperature such as toughness, strength, and elasticity. Conversely, biocomposites with weak thermal stability could evolve hazardous volatiles producing repulsive odors and would be highly unsuitable for indoor usage, for example, in construction and automobile parts.

**Figure 5:**
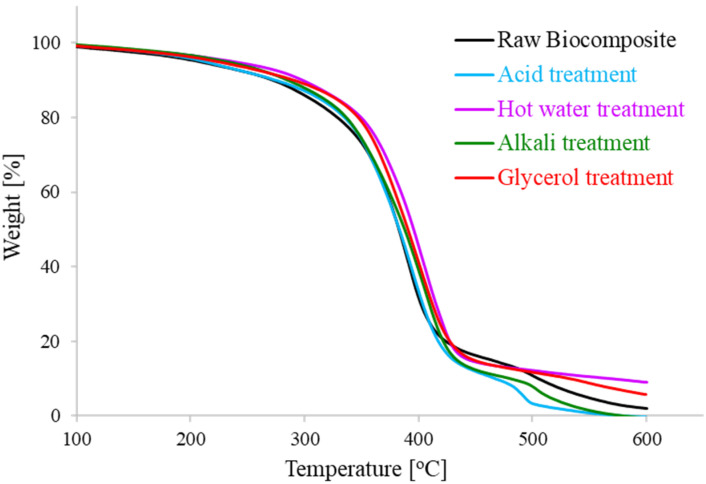
Thermal degradation profiles for untreated and treated biocomposite samples.

TGA profiles of composite sheets prepared using untreated and AAG, dilute acid, alkaline and hot water-pretreated WS biomass are shown in Figure 5 and Table 2 provides the various thermal parameters inferred from these profiles. The thermogravimetry results clearly imply that all biocomposites prepared from treated WS fibers exhibited superior thermal properties compared to untreated biocomposites. The prominent *T*_p_ values of 407 °C, 408.4 °C, and 409.6 °C were found for alkali-, glycerol-, and hot water-treated composites, respectively, compared to that of untreated biocomposite (392.9 °C). The minimum value of residue at 600 °C (i.e., 1.3%) was obtained for alkali-treated composites, which may be due to the least amount of lignin left in the alkali-treated fiber, as evident from Figure 1.

The present results are in accordance with the literature as various studies reported a boost in the thermal potency of lignocellulosic fiber based composites after different pretreatments. An increase in the *T*_max_ value from 347.1 °C to 363 °C was observed for particulate composites fabricated form oil palm trunk after pretreatment with hot water [33]. An alkali (5% NaOH solution) pretreatment of eucalyptus wood enhanced the *T*_10_ value from 238 °C for untreated composites to 249 °C for biocomposites made from treated fiber [34]. Rosa and co-workers [30] also noted the improvement in the thermal stability of coir fiber based biocomposites after numerous pretreatments including hot water, mercerization and bleaching. Pretreatment of WS fiber partially removed the non-cellulosic constituents during dissolution and regenerated the lignocellulosic material in various regents which might be the main reason for higher thermal stability of the treated biocomposites. The fiber with higher cellulosic contents could induce strong fiber–matrix interaction so that exceeding amount of thermal energy is required to break such bonding when thermally decomposed [35].

### 3.4. Comparison of Mechanical Properties of Biocomposite Board

**Figure 6:**
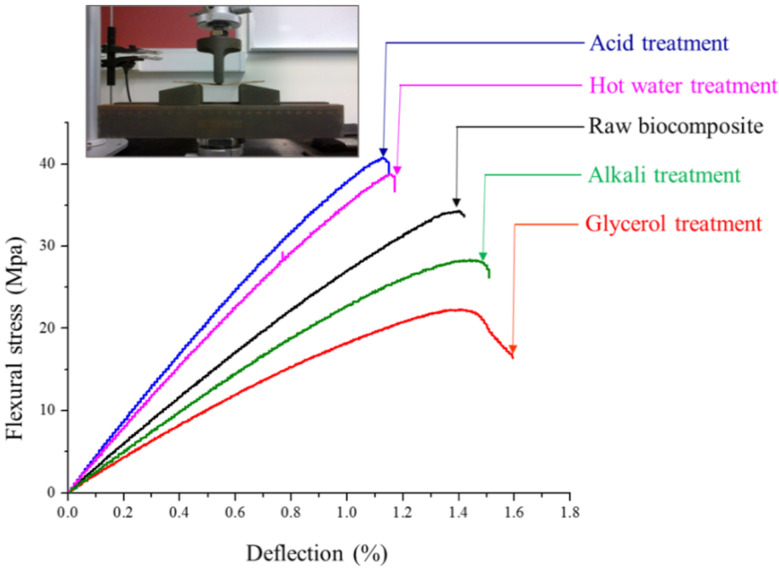
Flexural stress as a function of deflection for untreated and treated biocomposites.

Adequate mechanical characteristics of the biocomposites are mandatory for their widespread industrial usage in furniture, construction, and automotive sectors. Stress–strain profiles for the biocomposites made from raw and treated WS fibers are presented in Figure 6 and Table 3 summarizes different parameters conferred from these curves. Strain energy density (SED) is a useful material attribute and accounts for a measure of energy absorbed during flexural load and, hence, materials resistance to failure altogether. It is estimated from area under the stress–strain curve and, thus, represents overall mechanical characteristics including strength, elongation, and modulus [36]. Pretreatment of WS fiber significantly influenced the mechanical performance of the fabricated composite samples. The prominent strength and SED of 40.5 MPa and 24.15 MJ/m^3^, respectively, was noted for biocomposite sample prepared from acid treated WS fiber. Hot water pretreatment was also suited to manufacture the biocomposite sheets with elevated flexural strength and modulus, as evident from Table 3. However, pretreatment of WS fiber with glycerol and alkali solution negatively affect the mechanical properties of bicomposites and reduced the flexural strength to 22.22 MPa and 28.21 MPa, respectively, in comparison with the value of 33.57 MPa for untreated biocomposite. The values of the flexural modulus were also decreased from 2680.2 MPa for untreated biocomposite to 1810.4 MPa and 2427.2 MPa for glycerol and alkali treated biocomposites, respectively. 

The impact of different pretreatments of lignocellulosic fiber on the mechanical properties of biocomposites have been described in the literature and asserts the present results. Pretreatment of bagasse fiber with 1% sulfuric acid solution considerably enhanced the flexural strength of the fabricated composite sheets by 56% [37]. Increase in the modulus of rupture (MOR) of the coir fiber based biocomposites after hot water pretreatment was also reported [38]. Improved fiber–polymer interfacial conglutination due to elimination of surface impurities after pretreatment could be the prime impetus to enhance the flexural characteristics of the biocomposites [39]. Few studies described the slight improvement in the flexural properties of the biocomposites after alkaline pretreatment [40], nevertheless, significant reduction in the flexural characteristics of the composites after alkaline pretreatment of lignocellulosic fiber has also been reported [41]. Although alkaline and glycerol pretreatment increased the cellulosic contents of the WS fiber, but other parameters such as crystallinity, surface morphology, accessibility, pore structure, and polymorphic transformation to cellulose II from cellulose I also play crucial role in defining the final properties of the lignocellulosic fiber based composites [42]. Md. Saiful Islam et al. observed changes in the crystalline structure of lignocellulosic fiber as new peaks of different intensities were emerged in diffractograms of alkali pretreated fiber which improved the mechanical strength of the fabricated biocomposites. The formation of cellulose-ONa compound was also reported [43]. The acid pretreatment was found to deteriorate the mechanical properties of lignocellulosic rice particles. Indeed, the reason for the slight reduction in mechanical properties during alkali or glycerol pretreatment is not yet thoroughly understood, it might be due to physical changes including formation of more short fibers with lower length/diameter ratio during dissolution process [44]. Excessive leaching, fiber agglomeration or attrition, and damage of fibril structure could induce the weakened adhesion between fiber and polymer matrix and responsible to deteriorate the mechanical performance of the alkali and glycerol treated biocomposites. 

### 3.5. Comparison of Energy Consumption for Different WS Fiber Pretreatments

Total energy expenditure for biomass pretreatment is one of the critical factors to identify its economic feasibility. Energy consumed to process 1 kg of WS fiber by AAG, alkali, dilute acid, and hot water pretreatments is provided in Table 4. The energy needed for dilute alkali, acidic, and hot water solutions was almost similar having value of about 0.1285 kWh while energy consumption for glycerol pretreatment was 0.0850 kWh/kg of WS fiber. These calculations indicate that glycerol pretreatment consumed about 56% lower energy compared to other pretreatment processes. The comparison of energy expenditure for glycerol pretreatment with other technologies has not been reported yet in literature. However, Yoon et al. [24] claimed that the rice straw pretreatment with ionic liquid (i.e., 1-ethyl-3-methylimidazolium acetate) required about 55% lesser energy than that of acid and alkaline solutions. Energy required for biomass pretreatment mainly depends on the temperature and dissolution time, biomass loading, thermophysical characteristics of pretreated mixture and the flow pattern. Although stirring work required in case of glycerol pretreatment is higher compared to the other solutions (i.e., hot water, dilute acid and alkali) due to the high viscosity of glycerol but thermal energy required to conduct pretreatment at 130 °C is the major component of total pretreatment energy. The lower total energy consumption of glycerol pretreatment could be due to the lower specific heat and, hence, lower thermal energy expenditure of glycerol as compared to dilute acidic/alkaline reagents. The energy consumption for glycerol assisted pretreatment may further be improved by employing a higher solid loading as asserted in a sensitivity analysis based techno-economic study of biomass pretreatment process in biorefinery context [45]. Moreover, strategies to reduce viscosity of glycerol such as glycerol-water mixtures with promising capabilities to dissolve lignocellulose under mild operating conditions and modest processing time could also augment the comparative energy efficiency of glycerol based pretreatment process of lignocellulosic waste residues to manufacture biocomposite panels.

Organosolv processes using glycerol have been extensively studied to produce cellulose-rich fiber and fermentable sugars from lignocellulosic residues but commercialization of this technology for conversion of lignocellulose into green composites is currently confined. Some commercialized processes are being developed such as Organocell (Munich, Germany), Chempolis (Oulu, Finland), and glycerol-based Glycell™ (Leaf Resources, Queensland Australia); but considerably high pretreatment cost (e.g., for energy and solvent recovery) is still the main hurdle [46]. The energy consumption for glycerol assisted pretreatment processes for fabrication of composite panels can be further reduced by using a lower solvent-to-biomass ratio or by employing low cost co-solvents that can pretreat lignocellulose under relatively mild operating conditions and short dissolution time.

## 4. Conclusions

Sustainable biorefinery approach encourages the development of efficient pretreatment methods for lignocellulosic biomass and this study presents a comparative investigation for glycerol assisted pretreatment of wheat straw residue as a new approach to produce cellulose-rich fiber (CRF) for green composite fabrication. Lignocellulosic composition analysis of untreated and pretreated WS fibers enumerated the superiority of AAG pretreatment to produce CRF as compared to other pretreatment techniques such as hot water, dilute acid and alkaline solution. AAG pretreatment consumed about 56% lesser energy than other pretreatments indicating that this process could be favorable to operate at commercial scale. The composite samples prepared from dilute acid treated WS fibers showed an enhanced strain energy density (SED) of 24.15 MJ/m^3^ compared to a value of 23.85 MJ/m^3^ for untreated composites while the mechanical performance of the glycerol treated biocomposites was reduced with a SED value of 17.82 MJ/m^3^. Glycerol based processes could be a favorable technology for conversion of agri-waste into cellulosic fiber for green materials fabrication but uniform dispersion of treated fiber into polymer matrix to avoid agglomeration, limited knowledge regarding chemical reactivity of glycerol with solvated lignocellulosic components and optimization of pretreatment parameters are the captious issues need to be explored in the future.

## Figures and Tables

**Table 1:** Parameters derived from thermal profiles of treated and untreated WS fibers.

Fiber Samples	*T*_p_ (°C)	Weight Loss (%)	Residue Left at 600 °C (%)
Raw	297.2	90.8	9.1
Aqueous acidified glycerol (AAG) treated	338.4	95.0	4.9
Acid treated	315.8	94.0	5.9
Hot water treated	313.2	96.0	3.9
Alkali treated	333.1	96.5	3.4

**Table 2:** Parameters derived from thermal profiles of treated and untreated biocomposites.

Biocomposite Samples	*T*_p_ (°C)	Weight Loss (%)	Residue Left at 600 °C (%)
Raw	392.9	97.1	2.8
AAG treated	408.4	93.8	6.1
Acid treated	395.4	97.7	2.2
Hot water treated	409.6	90.0	9.9
Alkali treated	407.0	98.6	1.3

**Table 3:** Parameters derived from stress–strain profiles of untreated and treated biocomposites.

Biocomposite Samples	Flexural Modulus (MPa)	Flexural Strength (MPa)	Elongation at Break (%)	Strain Energy Density (MJ/m^3^)
Raw	2680.2 ± 85.3	33.57 ± 1.06	1.42 ± 0.007	23.85 ± 1.06
AAG treated	1810.4 ± 82.6	22.22 ± 1.01	1.66 ± 0.016	17.82 ± 1.01
Acid treated	4028.8 ± 44.2	40.50 ± 2.39	1.47 ± 0.19	24.15 ± 2.39
Hot water treated	3890.3 ± 108.1	38.71 ± 1.44	1.15 ± 0.12	22.23 ± 1.44
Alkali treated	2427.2 ± 64.8	28.21 ± 1.18	1.50 ± 0.09	22.65 ± 1.18

**Table 4:** Energy consumed for pretreatment of WS fiber by various processes.

Fiber Samples	Pretreatment Energy (kWh/kg of WS)
AAG treated	0.0850
Acid treated	0.1280
Hot water treated	0.1285
Alkali treated	0.1290

## Data Availability

The data presented in this study are available on request from the corresponding author.

## References

[1] Situmorang Y.A., Zhao Z., Yoshida A., Abudula A., Guan G. (2020). Small-scale biomass gasification systems for power generation (<200 kW class): A review. Renew. Sustain. Energy Rev..

[2] dos Santos M.F.N., Gomes Battistelle R.A., Bezerra B., Varum H. (2014). Comparative study of the life cycle assessment of particleboards made of residues from sugarcane bagasse (Saccharum spp.) and pine wood shavings (Pinus elliottii). J. Clean. Prod..

[3] Mahmood H., Moniruzzaman M., Yusup S., Akil H.M. (2018). Ionic liquid pretreatment at high solids loading: A clean approach for fabrication of renewable resource based particulate composites. Polym. Compos..

[4] Mahmood H., Moniruzzaman M., Iqubal T., Yusup S., Rashid M., Raza A. (2019). Comparative effect of ionic liquids pretreatment on thermogravimetric kinetics of crude oil palm biomass for possible sustainable exploitation. J. Mol. Liq..

[5] Behera S., Arora R., Nandgahopal N., Kumar S. (2014). Importance of chemical pretreatment for bioconversion of lignocellulosic biomass. Renew. Sustain. Energy Rev..

[6] Rajendran K., Drielak E., Varma V.S., Muthusamy S., Kumar G. (2018). Updates on the pretreatment of lignocellulosic feedstocks for bioenergy production—A review. Biomass Convers. Biorefinery.

[7] Charnnok B., Sakdaronnarong C., Sinbuathong N. (2019). Hydrothermal pretreatment with sulfonated bentonite catalyst enhances potassium removal and bioconversion of oil palm empty fruit bunch to sugar and biohydrogen. Biomass Convers. Biorefinery.

[8] Financie R., Moniruzzaman M., Uemura Y. (2016). Enhanced enzymatic delignification of oil palm biomass with ionic liquid pretreatment. Biochem. Eng. J..

[9] Zheng Y., Zhao J., Xu F., Li Y. (2014). Pretreatment of lignocellulosic biomass for enhanced biogas production. Prog. Energy Combust. Sci..

[10] Zadeh Z.E., Abdulkhani A., Aboelazayem O., Saha B. (2020). Recent insights into lignocellulosic biomass pyrolysis: A critical review on pretreatment, characterization, and products upgrading. Processes.

[11] Nazli Borand M.N., Karaosmanoglu F. (2018). Effects of organosolv pretreatment conditions for lignocellulosic biomass in biorefinery applications: A review. J. Renew. Sustain. Energy.

[12] Zhang K., Pei Z., Wang D. (2016). Organic solvent pretreatment of lignocellulosic biomass for biofuels and biochemicals: A review. Bioresour. Technol..

[13] Gu Y., Jerome F. (2010). Glycerol as a sustainable solvent for green chemistry. Green Chem..

[14] Ahmad A., Mahmood H., Mansor N., Iqbal I., Moniruzzaman M. (2020). Ionic liquid assisted polyetheretherketone-multiwalled carbon nanotubes nanocomposites: An environmentally friendly approach. J. Appl. Polym. Sci..

[15] Lynam J.G., Chow G.I., Hyland P.L., Coronella C.J. (2016). Corn stover pretreatment by ionic liquid and glycerol mixtures with their density, viscosity, and thermogravimetric properties. ACS Sustain. Chem. Eng..

[16] Sun F.F., Zhao X., Hong J., Tang Y., Wang L., Sun H., Xiang L., Hu J. (2016). Industrially relevant hydrolyzability and fermentability of sugarcane bagasse improved effectively by glycerol organosolv pretreatment. Biotechnol. Biofuels.

[17] Trinh L.T.P., Lee J.-W., Lee H.-J. (2016). Acidified glycerol pretreatment for enhanced ethanol production from rice straw. Biomass Bioenergy.

[18] Sun F., Wang L., Hong J., Ren J., Du F., Hu J., Zhang Z., Zhou B. (2015). The impact of glycerol organosolv pretreatment on the chemistry and enzymatic hydrolyzability of wheat straw. Bioresour. Technol..

[19] Romaní A., Ruiz H.A., Teixeira J.A., Domingues L. (2016). Valorization of eucalyptus wood by glycerol-organosolv pretreatment within the biorefinery concept: An integrated and intensified approach. Renew. Energy.

[20] Zhang Z., Wong H.H., Albertson P.L., Doherty W.O., O’Hara I.M. (2013). Laboratory and pilot scale pretreatment of sugarcane bagasse by acidified aqueous glycerol solutions. Bioresour. Technol..

[21] Yu H., Zhang M., Ouyang J., Shen Y. (2014). Comparative study on four chemical pretreatment methods for an efficient saccharification of corn stover. Energy Fuels.

[22] Felder R.M., Rousseau R.W., Bullard L.G. (1986). Elementary Principles of Chemical Processes.

[23] McCabe W.L., Smith J.C., Harriott P. (1993). Unit Operations of Chemical Engineering.

[24] Yoon L.W., Ngoh G.C., Chua A.S.M., Hashim M.A. (2011). Comparison of ionic liquid, acid and alkali pretreatments for sugarcane bagasse enzymatic saccharification. J. Chem. Technol. Biotechnol..

[25] Mahmood H., Moniruzzaman M., Yusup S., Muhammad N., Iqbal T., Akil H.M. (2017). Ionic liquids pretreatment for fabrication of agro-residue/thermoplastic starch based composites: A comparative study with other pretreatment technologies. J. Clean. Prod..

[26] Haque M., Hasan M., Islam S., Ali E. (2009). Physico-mechanical properties of chemically treated palm and coir fiber reinforced polypropylene composites. Bioresour. Technol..

[27] Srivaro S., Matan N., Lam F. (2015). Stiffness and strength of oil palm wood core sandwich panel under center point bending. Mater. Des..

[28] Dorez G., Taguet A., Ferry L., Lopez-Cuesta J.M. (2013). Thermal and fire behavior of natural fibers/PBS biocomposites. Polym. Degrad. Stab..

[29] Mahmood H., Moniruzzaman M., Yusup S., Akil H.M. (2016). Particulate composites based on ionic liquid-treated oil palm fiber and thermoplastic starch adhesive. Clean Technol. Environ. Policy.

[30] Rosa M.F., Chiou B.-S., Medeiros E.S., Wood D.F., Williams T.G., Mattoso L.H., Orts W.J., Imam S.H. (2009). Effect of fiber treatments on tensile and thermal properties of starch/ethylene vinyl alcohol copolymers/coir biocomposites. Bioresour. Technol..

[31] Vilaplana F., Strömberg E., Karlsson S. (2010). Environmental and resource aspects of sustainable biocomposites. Polym. Degrad. Stab..

[32] Yang H., Yan R., Chen H., Zheng C., Lee A.D.H., Liang D.T. (2006). In-depth investigation of biomass pyrolysis based on three major components: Hemicellulose, cellulose and lignin. Energy Fuels.

[33] Jumhuri N., Hashim R., Sulaiman O., Nadhari W.N.A.W., Salleh K.M., Khalid I., Saharudin N.I., Razali M.Z. (2014). Effect of treated particles on the properties of particleboard made from oil palm trunk. Mater. Des..

[34] Rojo E., Alonso M.V., Oliet M., Del Saz-Orozco B., Rodriguez F. (2015). Effect of fiber loading on the properties of treated cellulose fiber-reinforced phenolic composites. Compos. Part B Eng..

[35] Mahmood H., Moniruzzaman M., Yusup S., Welton T. (2017). Ionic liquids assisted processing of renewable resources for the fabrication of biodegradable composite materials. Green Chem..

[36] Huffman P.J., Ferreira J., Correia J., De Jesus A., Lesiuk G., Berto F., Fernandez-Canteli A., Glinka G. (2017). Fatigue crack propagation prediction of a pressure vessel mild steel based on a strain energy density model. Frat. Integrità Strutt..

[37] Vilay V., Mariatti M., Taib R.M., Todo M. (2008). Effect of fiber surface treatment and fiber loading on the properties of bagasse fiber-reinforced unsaturated polyester composites. Compos. Sci. Technol..

[38] Asasutjarit C., Charoenvai S., Hirunlabh J., Khedari J. (2009). Materials and mechanical properties of pretreated coir-based green composites. Compos. Part B Eng..

[39] Qian S., Mao H., Sheng K., Lu J., Luo Y., Hou C. (2013). Effect of low-concentration alkali solution pretreatment on the properties of bamboo particles reinforced poly (lactic acid) composites. J. Appl. Polym. Sci..

[40] Rout J., Misra M., Tripathy S., Nayak S., Mohanty A.K. (2001). The influence of fibre treatment on the performance of coir-polyester composites. Compos. Sci. Technol..

[41] Gu H. (2009). Tensile behaviours of the coir fibre and related composites after NaOH treatment. Mater. Des..

[42] Gupta P., Uniyal V., Naithani S. (2013). Polymorphic transformation of cellulose I to cellulose II by alkali pretreatment and urea as an additive. Carbohydr. Polym..

[43] Islam M.S., Hamdan S., Jusoh I., Rahman R., Ahmed A.S. (2012). The effect of alkali pretreatment on mechanical and morphological properties of tropical wood polymer composites. Mater. Des..

[44] Li X., Wua Y., Cai Z., Winandy J.E. (2013). Primary properties of MDF using thermomechanical pulp made from oxalic acid pretreated rice straw particles. Ind. Crop. Prod..

[45] Klein-Marcuschamer D., Simmons B.A., Blanch H.W. (2011). Techno-economic analysis of a lignocellulosic ethanol biorefinery with ionic liquid pre-treatment. Biofuels Bioprod. Biorefining.

[46] Zhu J., Pan X. (2010). Woody biomass pretreatment for cellulosic ethanol production: Technology and energy consumption evaluation. Bioresour. Technol..

